# Association of circulating tumor HPV16DNA levels and quantitative PET parameters in patients with HPV-positive head and neck squamous cell carcinoma

**DOI:** 10.1038/s41598-024-53894-4

**Published:** 2024-02-08

**Authors:** Mitsuaki Tatsumi, Hidenori Tanaka, Yukinori Takenaka, Motoyuki Suzuki, Takahito Fukusumi, Hirotaka Eguchi, Tadashi Watabe, Hiroki Kato, Shinichi Yachida, Hidenori Inohara, Noriyuki Tomiyama

**Affiliations:** 1 Department of Radiology, Osaka University Hospital, 2-2-D1 Yamadaoka, Suita, Osaka 565-0871 Japan; 2 Department of Otorhinolaryngology-Head and Neck Surgery, Osaka University Graduate School of Medicine, Suita, Osaka Japan; 3 Department of Nuclear Medicine, Osaka University Graduate School of Medicine, Suita, Osaka Japan; 4 Department of Cancer Genome Informatics, Osaka University Graduate School of Medicine, Suita, Osaka Japan; 5 Department of Radiology, Osaka University Graduate School of Medicine, Suita, Osaka Japan

**Keywords:** Biomarkers, Oncology

## Abstract

Circulating tumor DNA (ctDNA), which circulates in the blood after being shed from cancer cells in the body, has recently gained attention as an excellent tumor marker. The purpose of this study was to evaluate whether ct human papillomavirus (HPV) 16 DNA (ctHPV16DNA) levels were associated with quantitative PET parameters in patients with HPV-positive head and neck (HN) squamous cell carcinoma (SCC). Fifty patients with oropharyngeal SCC (OPSCC) and 5 with SCC of unknown primary (SCCUP) before treatment were included. They all underwent blood sampling to test ctHPV16DNA levels and FDG PET-CT examinations. Quantitative PET parameters included SUVmax, metabolic tumor volume (MTV), MTV of whole-body lesions (wbMTV), and 56 texture features. ctHPV16DNA levels were compared to texture features of primary tumors in OPSCC patients (Group A) or the largest primary or metastatic lymph node lesions in OPSCC and SCCUP patients (Group B) and to other PET parameters. Spearman rank correlation test and multiple regression analysis were used to confirm the associations between ctHPV16DNA levels and PET parameters. ctHPV16DNA levels moderately correlated with wbMTV, but not with SUVmax or MTV in Groups A and B. ctHPV16DNA levels exhibited a weak negative correlation with low gray-level zone emphasis in Groups A and B. Multiple regression analysis revealed that wbMTV and high gray-level zone emphasis were the significant factors for ctHPV16DNA levels in Group B. These results were not observed in Group A. This study demonstrated that ctHPV16DNA levels correlated with the whole-body tumor burden and tumor heterogeneity visualized on FDG PET-CT in patients with HPV-positive HNSCC.

## Introduction

Circulating tumor DNA (ctDNA), which circulates in the blood after being shed from cancer cells in the body, has recently gained attention as an excellent tumor marker to detect cancer at an early stage, monitor treatment response, and detect cancer recurrence^1,2^. The ctDNA test, also known as one of the liquid biopsies, provides quantitative information on gene expression in living tumors. Classical biopsy is invasive and the results depend on the samples or areas within the tumor, while the ctDNA test is almost non-invasive and reflects the whole-body tumor burden.

In clinical situations, medical imaging has long been used to assess the status of patients with malignancies. Medical imaging was originally developed as a tool for visual assessment, but recently its numerical information has been found to be valuable and its role as a biomarker is expected to increase^3^. Among others, 2-deoxy-2-[F-18]fluoro-D-glucose (FDG) positron emission tomography (PET) imaging has been reported to function as a valuable biomarker for disease activity^4^. Quantitative PET parameters, such as metabolic tumor volume (MTV) or texture features, have been used to predict treatment response^5–7^ or prognosis^8^ in patients with head and neck (HN) squamous cell carcinoma (SCC).

In this study, we evaluated whether ct human papillomavirus (HPV) 16 DNA (ctHPV16DNA) levels were associated with quantitative PET parameters in patients with HPV-positive HNSCC to demonstrate the quantitative connection between gene expression and imaging biomarkers. Whole-body FDG PET was used as the source of quantitative parameters as FDG uptake is closely related to tumor metabolic activity. In our previous study dealing with the diagnostic performance of oral HPV DNA, oral HPV mRNA, and ctHPVDNA in detecting HPV-positive oropharyngeal SCC (OPSCC) and SCC of unknown primary (SCCUP)^9^, the correlation was already reported between ctDNA levels and MTV obtained with FDG PET. The association between ctDNA levels and volumetric PET parameters has also been reported in several papers recently^10–12^. However, this study further explored the association between ctDNA and volumetric and other PET parameters in a larger number of patients than in our previous study.

## Materials and methods

### Patients

**Figure 1:**
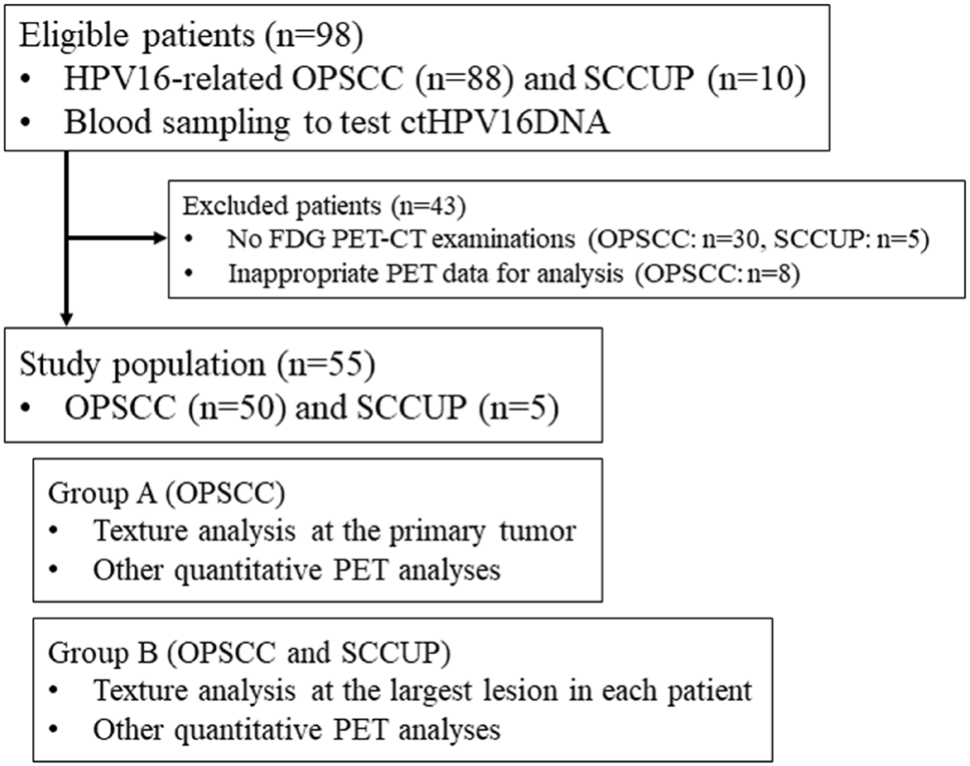
Flowchart of the patient enrollment.

This retrospective study was approved by Ethical Review Board Osaka University Hospital, which waived requirement for informed consent. Patients who were eligible for this study had been newly diagnosed as HPV16-related OPSCC (88 patients) and SCCUP (10 patients) between June 2017 and July 2021. HPV16 DNA was confirmed in these HPV16-related tumors by biopsy of the primary site for OPSCC and by fine needle aspiration or open biopsy of the neck for SCCUP. Details of the methods used for HPV16 DNA confirmation have been described elsewhere^13^. They all underwent venous blood sampling to test ctDNA levels. Among them, 58 patients with OPSCC and 5 with SCCUP underwent FDG PET-CT examinations. Eight patients with OPSCC were excluded from this study because of inappropriate PET data for analysis due to patient motion and the resulting incomplete image data quality. Finally, 50 patients with OPSCC and 5 with SCCUP were included in this study (Fig. 1). Patients' characteristics are shown in Table 1.

**Table 1:** Patients' characteristics.

	OPSCC (n = 50)	SCCUP (n = 5)
Sex		
Male/female	36/14	5/0
Age		
Range/median	42–93/67	53–68/55
Smoking history		
< 10 pack-years/≧10 pack-years	16/34	2/3
Primary subsite		
Lateral wall/anterior wall/superior wall/	38/10/1/	0/0/0/
Posterior wall/unknown	1/0	0/5
T classification		
T0/T1/T2/T3/T4	0/6/28/5/11	5/0/0/0/0
N classification		
N0/N1/N2/N3	4/31/11/4	0/2/2/1
M classification		
M0/M1	48/2	4/1
Stage		
I/II/III/IV	29/7/12/2	2/2/0/1

*OPSCC* Oropharyngeal squamous cell carcinoma, *SCCUP* Squamous cell carcinoma of unknown primary, *TNM* classification and Stage: Based on the eighth edition of UICC TNM classification system.

### Quantification of circulating tumor HPV16 DNA

Cell-free (cf) DNA was extracted from the plasma in patients with HPV16-related tumors, and absolute quantification of E6 and E7, the oncogenes of HPV16, in cfDNA, was then performed using the QX200 Droplet Digital PCR System (Bio-Rad, USA). ctHPV16DNA levels were obtained from the average of E6 and E7 and expressed as the number of HPV16 copies per mL of plasma.

### FDG PET-CT imaging

FDG PET-computed tomography (CT) imaging was performed with a GE Discovery 710 scanner equipped with a time-of-flight system. PET images from the top of skull to mid thighs were acquired for 2 min per one bed position 60 min after intravenous injection of FDG at a dose of 0.10 mCi/kg body weight. The PET transaxial field of view was 70 cm. They were reconstructed using OSEM (subset 8, iteration 3, and Gaussian filter 4 mm; regular setting in our hospital) as well as time-of-flight and point spread function. All PET images were reconstructed with a 192 × 192 matrix.

### Image analysis

Quantitative parameters in FDG PET-CT included the maximum value of standardized uptake value (SUVmax), MTV, and texture features of the primary tumor and the largest metastatic lymph node and MTV of whole-body lesions (wbMTV), which were obtained with commercially available software (PETSTAT: AdIn Research, Japan) in each patient.

SUVmax was obtained for quantitative metrics in PET. SUVmax was calculated from a single voxel exhibiting the maximum absolute SUV in each lesion. MTV was defined as the volume above the absolute SUV 2.5 threshold or the volume within a tumor margin delineated with 40% of SUVmax.

Volume of interests (VOIs) with the absolute SUV 2.5 threshold or the 40% of SUVmax threshold were placed semiautomatically on target lesions. VOIs were set not to include physiological FDG uptake. To extract the texture features, we equalized histograms by rescaling the intensity with 64 Gy levels between the absolute minimum and maximum values in each VOI. Fifty-six texture features were evaluated in this study, and entropy and homogeneity calculated from the co-occurrence matrix, low gray-level zone emphasis (LGZE) and high gray-level zone emphasis (HGZE) from the gray-level zone length matrix, and short-run emphasis (SRE) and long-run emphasis (LRE) from the gray-level run length matrix were included as recommended in the paper by Orlhac, et al.^14^. These six texture features, entropy, homogeneity, LGZE, HGZE, SRE, and LRE, were reported to be the most robust with respect to tumor region delineation and relatively independent from one another. Homogeneous lesions are known to have higher values of homogeneity, LGZE, and LRE and lower values of entropy, HGZE, and SRE than visually heterogeneous lesions^14^. All texture features used in this study were listed in the Supplemental file.

ctHPV16DNA levels were compared to texture features of primary tumors and other PET parameters in patients with OPSCC (Group A) or texture features of the largest lesions (primary tumors or metastatic lymph nodes) and other PET parameters in patients with OPSCC and SCCUP (Group B). Group A and B focused on the texture features of the primary OPSCC lesion and the largest lesion in each patient, respectively.

### Statistical analysis

Wilcoxon signed-rank test and Spearman rank correlation test were used to assess the difference and correlation, respectively, between MTV with the absolute SUV 2.5 threshold and MTV with the 40% of SUVmax threshold.

To remove redundant and unstable texture features, feature selection was performed using the Random Forest screening. Texture features with a contribution portion of more than 0.1 were included thereafter. SUVmax, MTV, and wbMTV were also assessed using the same method. Spearman rank correlation test and multiple regression analysis were used to evaluate the associations between ctHPV16DNA levels and PET quantitative parameters. Non-parametric tests were used because of the possible non-normal distribution of PET quantitative parameters.

All data were statistically analyzed using JMP Pro software (ver17.1.0, SAS Institute Inc., USA), and a p value less than 0.05 was considered statistically significant. Bonferroni corrections were applied to p values for multiple testing.

### Ethical approval

This study was performed in line with the principles of the Declaration of Helsinki. Institutional Review Board approval was obtained.

### Consent to participate

Written informed consent was waived by the Institutional Review Board.

## Results

### ctHPV16DNA levels

**Figure 2:**
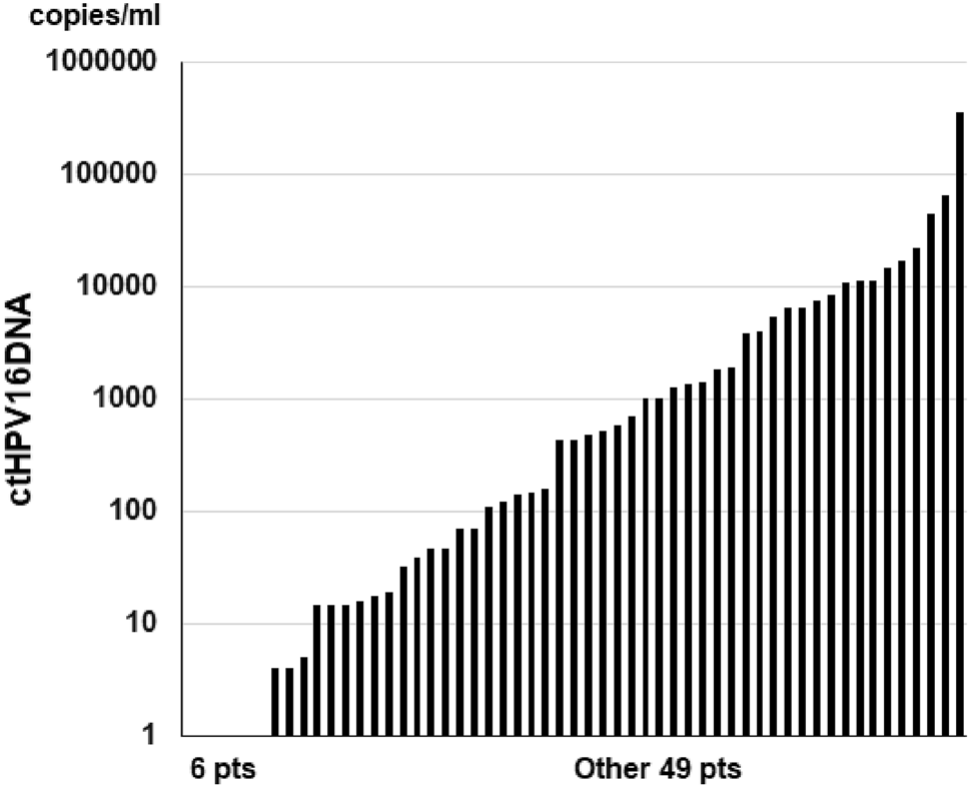
Distribution of ctHPV16DNA levels in all patients. The ctHPV16DNA level was 0 in 6 patients.

ctHPV16DNA levels ranged from 0 to 66,500 copies/mL with a median of 434.5 in Group A and from 0 to 364,944 copies/mL with a median of 435 in Group B. Six patients exhibited ctHPV16DNA of 0 copies/mL in both Groups A and B. The distribution of ctHPV16DNA levels in all patients is shown in Fig. 2.

### SUV-related and volumetric parameters in PET

Tumor FDG uptake was observed in all patients. SUVmax ranged from 6.7 to 28.3 with a mean of 15.4 ± 5.0 in Group A and from 6.7 to 28.6 with a mean of 15.9 ± 5.1 in Group B.

MTV with the absolute SUV 2.5 threshold was significantly larger than MTV with the 40% of SUVmax threshold in Groups A and B (*p* < 0.0001 for both), and they were highly correlated (Rho = 0.91 in Group A and 0.90 in Group B; *p* < 0.0001 for both). Thus, this study obtained MTV, wbMTV, and texture features in the tumors with the absolute SUV 2.5 threshold to avoid unreliable analysis due to small lesion sizes.

MTV with the absolute SUV 2.5 threshold ranged from 3.1 to 60.1 ml with a median of 17.7 in Group A and from 5.0 to 201.1 ml with a median of 20.1 in Group B. wbMTV with the absolute SUV 2.5 threshold ranged from 7.5 to 238.2 ml with a median of 30.7 in Group A and from 7.5 to 301.6 ml with a median of 29.8 in Group B.

### Association of ctDNA levels and quantitative PET parameters

In Group A, SUVmax MTV, wbMTV, and LGZE were selected as factors which had possible correlations with ctHPV16DNA. ctHPV16DNA levels correlated with wbMTV (Rho = 0.52, corrected *p* < 0.0005), but not with SUVmax or MTV. ctHPV16DNA levels exhibited a weak negative correlation with LGZE (Rho = − 0.35, corrected *p* < 0.05) among 56 texture features evaluated.

**Figure 3:**
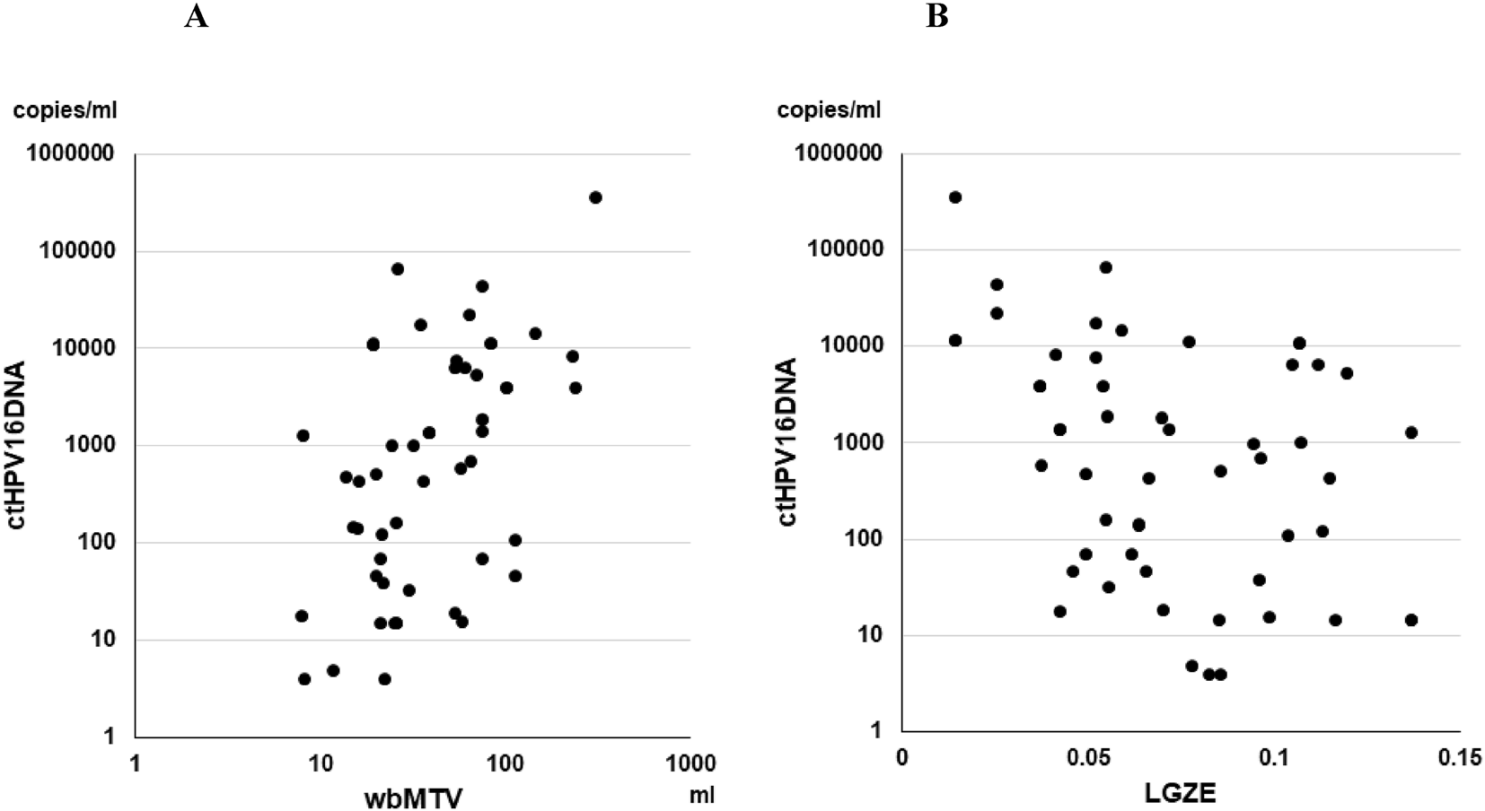
(**A** and **B**) The distribution of ctHPV16DNA levels versus wbMTV (2A) and LGZE (2B). ctHPV16DNA levels exhibited a moderately positive correlation with wbMTV (Rho = 0.53, *p* < 0.0005: 2A) and a weak negative correlation with LGZE (Rho =  − 0.36, *p* < 0.05: 2B). *wbMTV* whole-boy metabolic tumor volume, *LGZE* low gray-level zone emphasis

In Group B, SUVmax, wbMTV, HGZE, and LGZE were selected as factors which had possible correlations with ctHPV16DNA. The largest lesion in each patient was either a primary OPSCC lesion (40 patients) or a metastatic lymph node (15 patients; 10 from OPSCC and 5 from SCCUP). The results were similar to those in Group A. ctHPV16DNA levels correlated with wbMTV (Rho = 0.53, corrected *p* < 0.0005; Fig. 3A), but not with SUVmax or MTV. ctHPV16DNA levels exhibited a weak negative correlation with LGZE (Rho = − 0.36, corrected *p* < 0.05; Fig. 3B).

**Figure 4:**
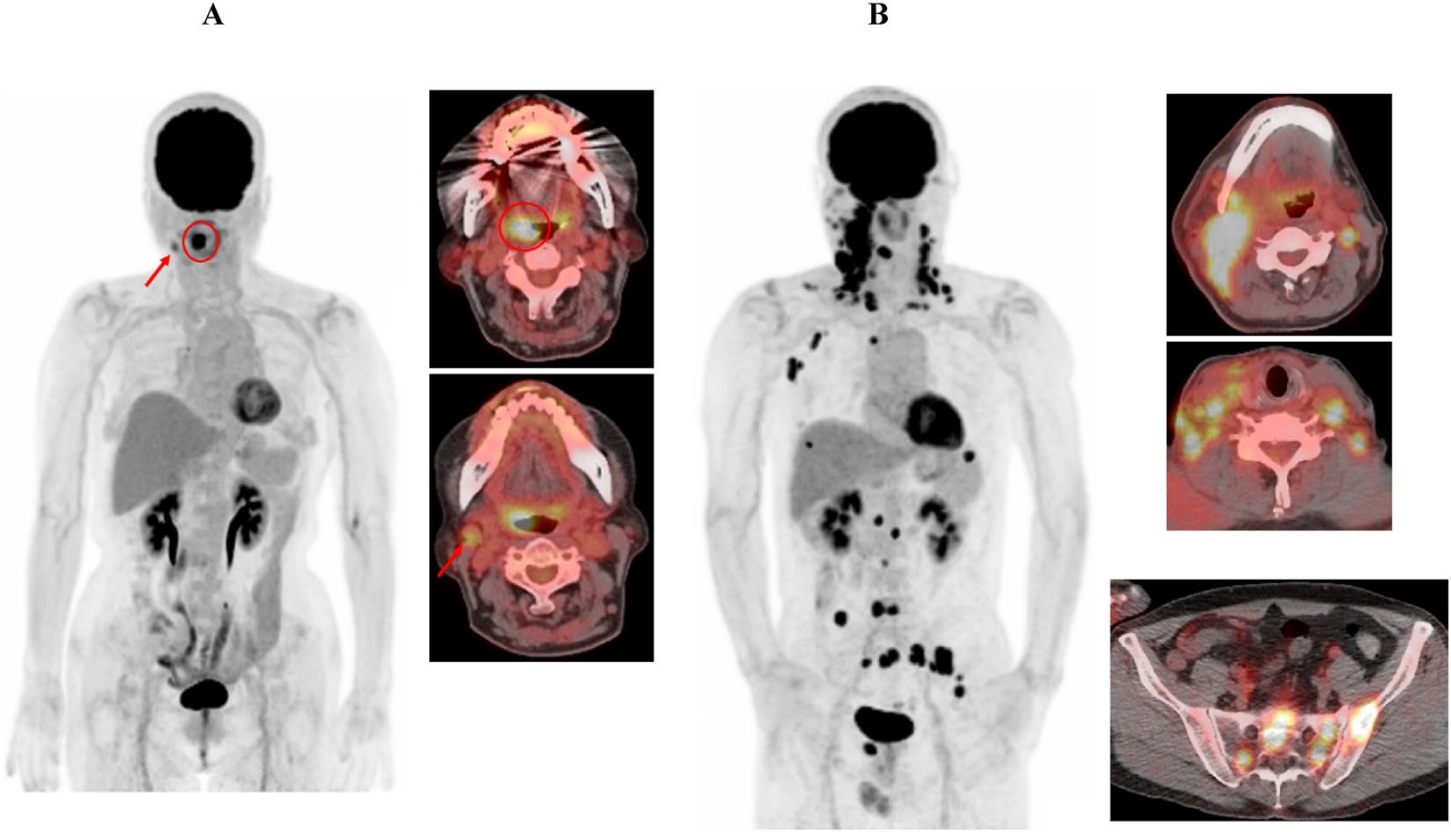
(**A**) A case with OPSCC and metastatic lymph node showing low ctHPV16DNA levels. Abnormal FDG uptake was observed in the right oropharyngeal (circles at the left and right upper images) and neck metastatic lymph node (arrows at the left and right lower images) lesions. ctHPV16DNA was 18 copies/ml and wbMTV was 7.81 ml. (**B**) A case with multiple metastatic lesions of SCCUP showing high ctHPV16DNA levels. Abnormal FDG uptake was observed throughout the body in the metastatic lesions of SCCUP, including neck (right upper), supraclavicular (right middle), and pelvic bone (right lower) lesions. ctHPV16DNA was 364,944 copies/ml and wbMTV was 301.61 ml.

**Figure 5:**
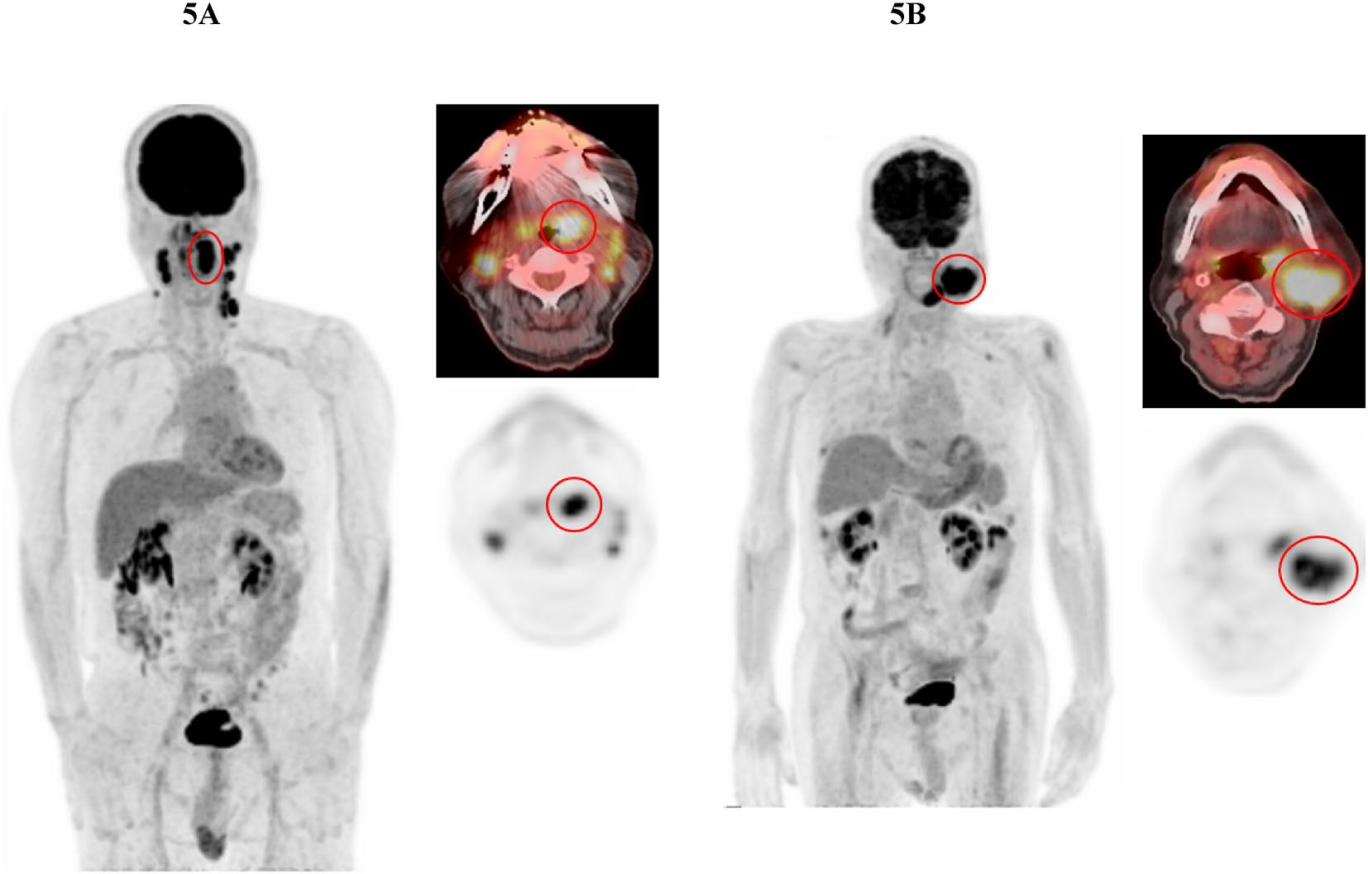
(**A** and **B**) Cases with homogeneous (4A) and heterogeneous (4B) FDG uptake in the largest SCC lesions. ctHPV16DNA was 5367 and 22,384 copies/ml, wbMTV was 68.81 and 63.42 ml, and LGZE was 0.1192 and 0.0251 in the case with homogeneous and heterogeneous FDG uptake, respectively.

Representative cases with low and high ctHPV16DNA levels showing low and high wbMTV values (tumor burden) are presented in Fig. 4A,B, respectively. Cases with homogeneous and heterogeneous FDG uptake in the largest SCC lesions are also presented in Fig. 5A,B, respectively. Higher ctHPV16DNA values were observed in the case with heterogeneous FDG uptake (low LGZE), although its wbMTV value was similar to the case with homogeneous FDG uptake (high LGZE).

The parameter ctHPV16DNA values divided by wbMTV was calculated to reduce the effect of tumor volume. ctHPV16DNA/wbMTV did not correlate with texture features in Groups A and B, but had a negative trend with LGZE (Rho = − 0.29, *p* = 0.13 [*p* < 0.05 before correction]) in Group B. Multiple regression analysis revealed that wbMTV (*p* < 0.0001, Standardized β: 0.66) and HGZE (*p* = 0.0025, Standardized β: 0.40) were the significant factors for ctHPV16DNA levels in Group B. These results were not obtained in Group A.

## Discussion

This study demonstrated the correlation between ctHPV16DNA levels and wbMTV in patients with HPV-positive HNSCC before treatment. Although the performance of ctHPV16DNA detection compared to FDG PET-CT in monitoring of treatment response has been evaluated and the superiority of ctHPV16DNA has been reported in previous studies^13,15^, this study presented data in the pretreatment period, which is still limited in reporting. Silvoniemi et al.^16^ recently reported an association between maximum variant allele frequency in the venous ctDNA samples and volumetric FDG PET parameters in HNSCC patients. They also presented pretreatment data while including p16-negative patients and evaluating total lesion glycolysis (MTV multiplied by SUVmean).

Tumor FDG uptake and wbMTV have been reported to reflect disease activity and active tumor burden in the body, respectively^17,18^. The association between ctDNA levels and wbMTV similar to this study has been reported in several papers recently^10–12^, and is an extremely significant finding in terms of the clear evidence of quantitative connection between gene expression and imaging biomarkers in living humans.

Pascual et al.^19^ reported that the measurement of low ctDNA expression was relatively unreliable because of the short half-lives of ctDNA and the sensitivity of the measurement method. On the other hand, even with an extremely high sensitivity PET system, lesions with a 10 mm diameter are not necessarily detected^20^. Although six patients had ctHPV16DNA of 0 in this study, superior detection of small lesions is generally expected with ctDNA as shown in another study showing more false-negative findings in PET than ctHPV16DNA^13^. Further technological improvements in ctDNA measurement as well as PET imaging are required to recognize the association of these measured values accurately and manage small lesions properly.

Another major finding in this study is that ctHPV16DNA levels correlated with intratumoral heterogeneity in the primary or the largest SCC lesions observed as LGZE of texture features among quantitative PET parameters. The parameter ctHPV16DNA/wbMTV to reduce the effect of tumor volume also correlated with intratumoral heterogeneity. These results indicated that higher ctHPV16DNA levels were expected in cases having heterogeneous than homogeneous lesions if wbMTV values were similar. Although no reports have been available regarding the association between ctDNA levels and intratumoral heterogeneity on PET, two papers have been published so far on CT^21,22^. Gill et al.^21^ demonstrated an association between ctDNA levels and intratumoral heterogeneity on CT in patients with metastatic melanoma. Although they measured the circulating tumor DNA mutant allele fraction for ctDNA, the association existed even when controlling for tumor volume. Lafata et al.^22^ reported an association between ctDNA levels and intratumoral heterogeneity on CT in patients with locally advanced lung cancer. The homogeneous attenuation pattern on CT correlated with ctDNA TP53 mutations in their study. Although its influence on ctDNA is considered smaller than wbMTV, intratumoral heterogeneity is also a valuable tumor characteristic associated with treatment response and outcome in various types of malignancies^23^.

The association between ctDNA levels and tumor burden or intratumoral heterogeneity was reported using surgical specimens in gastrointestinal stromal tumors^24^, however, this study successfully demonstrated the association using PET imaging technique in living HNSCC tumors. ctDNA has an advantage in detecting tiny lesions, while PET imaging is useful for visualizing and quantifying tumor activity. Knowing that ctDNA test and PET imaging are complementary, this study warrants that the combination of ctHPV16DNA and quantitative PET parameters is indispensable and a powerful method for the precision medicine in patients with HPV-positive HNSCC.

There were limitations in this study. This study was conducted retrospectively, and the number of patients analyzed was relatively small. As this study shows promise in using the combination of ctHPV16DNA and quantitative PET parameters, further studies are required to determine the clinical relevance of this combination in a larger number of patients. Another limitation was that this study was conducted exclusively in patients with HPV-positive HNSCC. The association between ctDNA levels and quantitative PET parameters in HNSCC and other malignancies in the pre- and post-treatment periods should be clarified with further accumulated data.

## Conclusion

This study demonstrated that ctHPV16DNA levels correlated with the whole-body tumor burden visualized on FDG PET-CT in patients with HPV-positive HNSCC. ctHPV16DNA levels exhibited a weak correlation with tumor heterogeneity, especially in large OPSCC or metastatic lymph node lesions.

### Supplementary Information

Supplementary Information.

## Data Availability

The datasets analyzed in during the current study are available from the corresponding author on reasonable request.
