# Supplementary material for: Association of circulating tumor HPV16DNA levels and quantitative PET parameters in patients with HPV-positive head and neck squamous cell carcinoma
Source: Sci Rep. 2024 Feb 8;14:3278. doi: 10.1038/s41598-024-53894-4 (PMC10853198; doi:10.1038/s41598-024-53894-4)
Supplement: Supplementary file 1 — Supplementary Information. [file 41598_2024_53894_MOESM1_ESM.docx]

**Texture features used in this study**

| SUV Histogram | Variance |
| --- | --- |
|  | Entropy |
| NGLCM | Uniformity mean |
|  | Uniformity max |
|  | Entropy mean |
|  | Entropy max |
|  | Dissimilarity mean |
|  | Dissimilarity max |
|  | Contrast mean |
|  | Contrast max |
|  | Homogeneity mean |
|  | Homogeneity max |
|  | Inverse Difference Moment mean |
|  | Inverse Difference Moment max |
|  | Correlation mean |
|  | Correlation max |
| NGTDM | Coarseness |
|  | Contrast |
|  | Busyness |
|  | Complexity |
|  | Strength |
| NGLCM3D | Uniformity mean |
|  | Uniformity max |
|  | Entropy mean |
|  | Entropy max |
|  | Dissimilarity mean |
|  | Dissimilarity max |
|  | Contrast mean |
|  | Contrast max |
|  | Homogeneity mean |
|  | Homogeneity max |
|  | Correlation mean |
|  | Correlation max |
| NGLCM3DMean | Uniformity |
|  | Entropy |
|  | Dissimilarity |
|  | Contrast |
|  | Homogeneity |
|  | Correlation |
| GLSZM | HighGrayLevelZoneEmphasis |
|  | LowGrayLevelZoneEmphasis |
|  | LargeAreaEmphasis |
|  | SmallAreaEmphasis |
|  | IntensityVariability |
|  | RunLengthVariability |
|  | ZonePercentage |
|  | ShortRunsEmphasis mean |
|  | ShortRunsEmphasis max |
|  | LongRunsEmphasis mean |
|  | LongRunsEmphasis max |
|  | GrayLevelNonuniformity mean |
|  | GrayLevelNonuniformity max |
|  | RunLengthNonuniformity mean |
| GLRLM | RunLengthNonuniformity max |
|  | RunPercentage mean |
|  | RunPercentage max |

NGLCM: Normalized Gray Level Cooccurrence Matrix

NGTDM: Neighborhood Gray Tone Difference Matrix

NGLCM3D: 3D Normalized Gray Level Cooccurrence Matrix

GLSZM: Gray Level Size Zone Matrix

GLRLM: Gray Level Run Length Matrix

**Random Forest screening to select possible variables**

Group A

SUVmax, MTV, wbMTV, and LowGrayLevelZoneEmphasis were selected as follows (contribution portion of more than 0.1).

| Predictor | Contribution | Portion | Rank |  |
| --- | --- | --- | --- | --- |
| **SUVmax** | **5.07E+08** | **0.3462** | **1** | **Selected** |
| **wbMTV** | **5.02E+08** | **0.3426** | **2** | **Selected** |
| **MTV** | **4.56E+08** | **0.3111** | **3** | **Selected** |
| **GLSZM** **LowGrayLevelZoneEmphasis** | **8.96E+08** | **0.476** | **1** | **Selected** |
| GLSZM RunLengthVariability | 1.08E+08 | 0.0572 | 2 |  |
| NGTDM Coarseness | 82683877 | 0.0439 | 3 |  |
| NGTDM Strength | 60490410 | 0.0321 | 4 |  |
| GLSZM HighGrayLevelZoneEmphasis | 54399723 | 0.0289 | 5 |  |
| NGLCM3D Homogeneity mean | 51613114 | 0.0274 | 6 |  |
| NGLCM3D Entropy max | 50093701 | 0.0266 | 7 |  |
| SUV Histogram Entropy | 49636400 | 0.0264 | 8 |  |
| NGTDM Complexity | 46138666 | 0.0245 | 9 |  |
| NGLCM3DMean Entropy | 42358809 | 0.0225 | 10 |  |
| NGLCM3D Correlation max | 39735397 | 0.0211 | 11 |  |
| SUV Histogram Variance | 33932609 | 0.018 | 12 |  |
| NGTDM Contrast | 32627333 | 0.0173 | 13 |  |
| GLSZM IntensityVariability | 29638134 | 0.0158 | 14 |  |
| NGTDM Busyness | 23923941 | 0.0127 | 15 |  |
| NGLCM3D Correlation mean | 21129275 | 0.0112 | 16 |  |
| NGLCM Uniformity max | 20065394 | 0.0107 | 17 |  |
| NGLCM Homogeneity max | 19451049 | 0.0103 | 18 |  |
| NGLCM Homogeneity mean | 18273468 | 0.0097 | 19 |  |
| NGLCM Correlation mean | 17619815 | 0.0094 | 20 |  |
| NGLCM3D Uniformity max | 16504703 | 0.0088 | 21 |  |
| GLRLM ShortRunsEmphasis max | 13878491 | 0.0074 | 22 |  |
| NGLCM Uniformity mean | 13286165 | 0.0071 | 23 |  |
| GLRLM RunPercentage max | 12610002 | 0.0067 | 24 |  |
| NGLCM Correlation max | 11674305 | 0.0062 | 25 |  |
| NGLCM3D Uniformity mean | 11082384 | 0.0059 | 26 |  |
| NGLCM3DMean Uniformity | 10110573 | 0.0054 | 27 |  |
| NGLCM3DMean Contrast | 9364376 | 0.005 | 28 |  |
| GLRLM LongRunsEmphasis max | 8128341 | 0.0043 | 29 |  |
| NGLCM3D Entropy mean | 7329664 | 0.0039 | 30 |  |
| GLRLM GrayLevelNonuniformity max | 6979369 | 0.0037 | 31 |  |
| NGLCM3DMean Dissimilarity | 6560511 | 0.0035 | 32 |  |
| NGLCM Dissimilarity max | 6293854 | 0.0033 | 33 |  |
| NGLCM Inverse Difference Moment max | 5756465 | 0.0031 | 34 |  |
| NGLCM3D Contrast max | 5442148 | 0.0029 | 35 |  |
| NGLCM3D Homogeneity max | 5129118 | 0.0027 | 36 |  |
| GLRLM RunLengthNonuniformity mean | 4590634 | 0.0024 | 37 |  |
| GLSZM LargeAreaEmphasis | 4414213 | 0.0023 | 38 |  |
| NGLCM3D Dissimilarity max | 3324777 | 0.0018 | 39 |  |
| NGLCM Contrast mean | 2970922 | 0.0016 | 40 |  |
| NGLCM Contrast max | 2613866 | 0.0014 | 41 |  |
| NGLCM Inverse Difference Moment mean | 2503338 | 0.0013 | 42 |  |
| GLRLM GrayLevelNonuniformity mean | 2248304 | 0.0012 | 43 |  |
| NGLCM3D Contrast mean | 1867184 | 0.001 | 44 |  |
| NGLCM Dissimilarity mean | 1820334 | 0.001 | 45 |  |
| GLRLM RunLengthNonuniformity max | 1811826 | 0.001 | 46 |  |
| GLSZM SmallAreaEmphasis | 1695560 | 0.0009 | 47 |  |
| GLSZM ZonePercentage | 1610874 | 0.0009 | 48 |  |
| NGLCM3DMean Homogeneity | 1073024 | 0.0006 | 49 |  |
| NGLCM3DMean Correlation | 722971.7 | 0.0004 | 50 |  |
| NGLCM Entropy max | 393166 | 0.0002 | 51 |  |
| NGLCM Entropy mean | 317622.4 | 0.0002 | 52 |  |
| NGLCM3D Dissimilarity mean | 308787.3 | 0.0002 | 53 |  |
| GLRLM ShortRunsEmphasis mean | 106805.4 | 0.0001 | 54 |  |
| GLRLM LongRunsEmphasis mean | 37390.38 | 0 | 55 |  |
| GLRLM RunPercentage mean | 0.7225 | 0 | 56 |  |

Group B

SUVmax, wbMTV, HighGrayLevelZoneEmphasis, and LowGrayLevelZoneEmphasis were selected as follows (contribution portion of more than 0.1).

| Predictor | | Contribution | | Portion | | Rank | |  | |
| --- | --- | --- | --- | --- | --- | --- | --- | --- | --- |
| **wbMTV** | | **1.60E+10** | | **0.7312** | | **1** | | **Selected** | |
| **SUVmax** | | **4.59E+09** | | **0.2099** | | **2** | | **Selected** | |
| MTV | | 1.29E+09 | | 0.0589 | | 3 | |  | |
| **GLSZM LowGrayLevelZoneEmphasis** | **1.22E+10** | | **0.3948** | | **1** | | **Selected** | |  |
| **GLSZM HighGrayLevelZoneEmphasis** | | **5.37E+09** | | **0.1745** | | **2** | | **Selected** | |
| GLRLM GrayLevelNonuniformity max | | 3.05E+09 | | 0.0991 | | 3 | |  | |
| GLSZM IntensityVariability | | 3.05E+09 | | 0.099 | | 4 | |  | |
| GLSZM RunLengthVariability | | 2.23E+09 | | 0.0723 | | 5 | |  | |
| GLRLM GrayLevelNonuniformity mean | | 1.97E+09 | | 0.0639 | | 6 | |  | |
| GLRLM RunLengthNonuniformity max | | 1.87E+09 | | 0.0608 | | 7 | |  | |
| GLRLM RunLengthNonuniformity mean | | 4.7E+08 | | 0.0153 | | 8 | |  | |
| NGLCM3D Homogeneity mean | | 94771543 | | 0.0031 | | 9 | |  | |
| SUV Histogram Entropy | | 80771872 | | 0.0026 | | 10 | |  | |
| SUV Histogram Variance | | 72533004 | | 0.0024 | | 11 | |  | |
| NGLCM Contrast mean | | 57074727 | | 0.0019 | | 12 | |  | |
| NGLCM3D Contrast mean | | 56388333 | | 0.0018 | | 13 | |  | |
| GLRLM LongRunsEmphasis max | | 30355306 | | 0.001 | | 14 | |  | |
| NGLCM3D Uniformity max | | 20740973 | | 0.0007 | | 15 | |  | |
| NGLCM3DMean Entropy | | 19701600 | | 0.0006 | | 16 | |  | |
| NGLCM3D Dissimilarity mean | | 15209129 | | 0.0005 | | 17 | |  | |
| NGLCM Homogeneity max | | 13834023 | | 0.0004 | | 18 | |  | |
| NGTDM Strength | | 13402059 | | 0.0004 | | 19 | |  | |
| NGLCM Contrast max | | 13085015 | | 0.0004 | | 20 | |  | |
| NGLCM Uniformity max | | 12877859 | | 0.0004 | | 21 | |  | |
| NGLCM Uniformity mean | | 11524412 | | 0.0004 | | 22 | |  | |
| GLSZM SmallAreaEmphasis | | 11518051 | | 0.0004 | | 23 | |  | |
| NGLCM Homogeneity mean | | 11104949 | | 0.0004 | | 24 | |  | |
| NGLCM3D Uniformity mean | | 8034354 | | 0.0003 | | 25 | |  | |
| NGLCM3D Homogeneity max | | 7445964 | | 0.0002 | | 26 | |  | |
| NGLCM Correlation max | | 7343945 | | 0.0002 | | 27 | |  | |
| NGLCM Inverse Difference Moment max | | 7262579 | | 0.0002 | | 28 | |  | |
| GLRLM RunPercentage max | | 7123695 | | 0.0002 | | 29 | |  | |
| GLRLM ShortRunsEmphasis mean | | 6237855 | | 0.0002 | | 30 | |  | |
| GLSZM LargeAreaEmphasis | | 5127241 | | 0.0002 | | 31 | |  | |
| NGTDM Coarseness | | 4433363 | | 0.0001 | | 32 | |  | |
| NGLCM3DMean Homogeneity | | 3937038 | | 0.0001 | | 33 | |  | |
| NGLCM3D Dissimilarity max | | 3709859 | | 0.0001 | | 34 | |  | |
| NGLCM3DMean Dissimilarity | | 3416910 | | 0.0001 | | 35 | |  | |
| NGTDM Contrast | | 3150569 | | 0.0001 | | 36 | |  | |
| GLRLM ShortRunsEmphasis max | | 3038586 | | 0.0001 | | 37 | |  | |
| NGLCM Inverse Difference Moment mean | | 2835136 | | 0.0001 | | 38 | |  | |
| NGLCM Entropy max | | 2483182 | | 0.0001 | | 39 | |  | |
| NGLCM3DMean Uniformity | | 2318642 | | 0.0001 | | 40 | |  | |
| GLSZM ZonePercentage | | 2059089 | | 0.0001 | | 41 | |  | |
| NGLCM3DMean Contrast | | 1999203 | | 0.0001 | | 42 | |  | |
| NGLCM3D Entropy mean | | 1638554 | | 0.0001 | | 43 | |  | |
| NGLCM3D Correlation mean | | 1196196 | | 0 | | 44 | |  | |
| NGLCM Correlation mean | | 1118008 | | 0 | | 45 | |  | |
| NGTDM Busyness | | 1009205 | | 0 | | 46 | |  | |
| NGLCM3D Correlation max | | 814409.6 | | 0 | | 47 | |  | |
| NGLCM Entropy mean | | 633334.1 | | 0 | | 48 | |  | |
| NGLCM Dissimilarity mean | | 604167.2 | | 0 | | 49 | |  | |
| NGLCM3D Entropy max | | 571974.5 | | 0 | | 50 | |  | |
| NGLCM3D Contrast max | | 567787.8 | | 0 | | 51 | |  | |
| NGLCM3DMean Correlation | | 455676.1 | | 0 | | 52 | |  | |
| GLRLM LongRunsEmphasis mean | | 401095.5 | | 0 | | 53 | |  | |
| NGTDM Complexity | | 39509.64 | | 0 | | 54 | |  | |
| NGLCM Dissimilarity max | | 33486.52 | | 0 | | 55 | |  | |
| GLRLM RunPercentage mean | | 5985.333 | | 0 | | 56 | |  | |

**Spearman rank correlation test**

Group A

ctHPV16DNA vs. Variable

| Variable | Spearman Rho | p value | Corrected p value |
| --- | --- | --- | --- |
| **SUVmax** | **-0.1011** | **0.4847** | **1.9388** |
| **MTV** | **0.1428** | **0.3225** | **1.29** |
| **wbMTV** | **0.5178** | **0.0001** | **0.0004** |
| SUV Histogram Variance | 0.0246 | 0.8653 |  |
| SUV Histogram Entropy | 0.2145 | 0.1347 |  |
| NGLCM Uniformity mean | -0.1914 | 0.1831 |  |
| NGLCM Uniformity max | -0.2091 | 0.145 |  |
| NGLCM Entropy mean | 0.1665 | 0.2478 |  |
| NGLCM Entropy max | 0.1526 | 0.29 |  |
| NGLCM Dissimilarity mean | -0.1912 | 0.1836 |  |
| NGLCM Dissimilarity max | -0.2225 | 0.1205 |  |
| NGLCM Contrast mean | -0.2587 | 0.0697 |  |
| NGLCM Contrast max | -0.2664 | 0.0615 |  |
| NGLCM Homogeneity mean | 0.0815 | 0.5737 |  |
| NGLCM Homogeneity max | 0.0189 | 0.8961 |  |
| NGLCM Inverse Difference Moment mean | 0.0502 | 0.729 |  |
| NGLCM Inverse Difference Moment max | 0.0408 | 0.7787 |  |
| NGLCM Correlation mean | -0.1915 | 0.1827 |  |
| NGLCM Correlation max | -0.2063 | 0.1506 |  |
| NGTDM Coarseness | -0.1662 | 0.2486 |  |
| NGTDM Contrast | -0.2072 | 0.1488 |  |
| NGTDM Busyness | 0.1703 | 0.237 |  |
| NGTDM Complexity | -0.1754 | 0.2231 |  |
| NGTDM Strength | -0.1255 | 0.3853 |  |
| NGLCM3D Uniformity mean | -0.186 | 0.196 |  |
| NGLCM3D Uniformity max | -0.171 | 0.2352 |  |
| NGLCM3D Entropy mean | 0.1668 | 0.247 |  |
| NGLCM3D Entropy max | 0.1595 | 0.2686 |  |
| NGLCM3D Dissimilarity mean | -0.1733 | 0.2288 |  |
| NGLCM3D Dissimilarity max | -0.0878 | 0.5442 |  |
| NGLCM3D Contrast mean | -0.2116 | 0.1401 |  |
| NGLCM3D Contrast max | -0.1141 | 0.4303 |  |
| NGLCM3D Homogeneity mean | 0.0809 | 0.5763 |  |
| NGLCM3D Homogeneity max | 0.0003 | 0.9981 |  |
| NGLCM3D Correlation mean | -0.1903 | 0.1855 |  |
| NGLCM3D Correlation max | -0.1935 | 0.1781 |  |
| NGLCM3DMean Uniformity | -0.1454 | 0.3136 |  |
| NGLCM3DMean Entropy | 0.1893 | 0.188 |  |
| NGLCM3DMean Dissimilarity | -0.2027 | 0.158 |  |
| NGLCM3DMean Contrast | -0.2421 | 0.0903 |  |
| NGLCM3DMean Homogeneity | 0.0875 | 0.5459 |  |
| NGLCM3DMean Correlation | -0.2008 | 0.1621 |  |
| GLSZM HighGrayLevelZoneEmphasis | 0.1433 | 0.3208 |  |
| **GLSZM LowGrayLevelZoneEmphasis** | **-0.3516** | **0.0123** | **0.0492** |
| GLSZM LargeAreaEmphasis | 0.079 | 0.5857 |  |
| GLSZM SmallAreaEmphasis | -0.0827 | 0.5679 |  |
| GLSZM IntensityVariability | -0.0456 | 0.7531 |  |
| GLSZM RunLengthVariability | 0.0188 | 0.8967 |  |
| GLSZM ZonePercentage | -0.079 | 0.5857 |  |
| GLRLM ShortRunsEmphasis mean | -0.0353 | 0.8075 |  |
| GLRLM ShortRunsEmphasis max | -0.0552 | 0.7033 |  |
| GLRLM LongRunsEmphasis mean | 0.0828 | 0.5675 |  |
| GLRLM LongRunsEmphasis max | 0.1151 | 0.4262 |  |
| GLRLM GrayLevelNonuniformity mean | -0.0278 | 0.8479 |  |
| GLRLM GrayLevelNonuniformity max | -0.0277 | 0.8486 |  |
| GLRLM RunLengthNonuniformity mean | 0.005 | 0.9725 |  |
| GLRLM RunLengthNonuniformity max | -0.0085 | 0.9535 |  |
| GLRLM RunPercentage mean | -0.0896 | 0.5358 |  |
| GLRLM RunPercentage max | -0.0851 | 0.5569 |  |

Group B

ctHPV16DNA vs. Variable

| Variable | Spearman Rho | p value | Corrected p value |
| --- | --- | --- | --- |
| **SUVmax** | **0.0978** | **0.4774** | **1.9096** |
| MTV | 0.2901 | 0.0317 |  |
| **wbMTV** | **0.526** | **<0.0001** | **<0.0004** |
| SUV Histogram Variance | -0.1839 | 0.179 |  |
| SUV Histogram Entropy | 0.2557 | 0.0595 |  |
| NGLCM Uniformity mean | -0.2219 | 0.1035 |  |
| NGLCM Uniformity max | -0.2199 | 0.1067 |  |
| NGLCM Entropy mean | 0.1551 | 0.2583 |  |
| NGLCM Entropy max | 0.1403 | 0.3068 |  |
| NGLCM Dissimilarity mean | -0.2469 | 0.0692 |  |
| NGLCM Dissimilarity max | -0.2045 | 0.1343 |  |
| NGLCM Contrast mean | -0.2909 | 0.0312 |  |
| NGLCM Contrast max | -0.2511 | 0.0644 |  |
| NGLCM Homogeneity mean | 0.1557 | 0.2562 |  |
| NGLCM Homogeneity max | 0.1432 | 0.2969 |  |
| NGLCM Inverse Difference Moment mean | 0.1245 | 0.3652 |  |
| NGLCM Inverse Difference Moment max | 0.1523 | 0.2669 |  |
| NGLCM Correlation mean | -0.2462 | 0.07 |  |
| NGLCM Correlation max | -0.2604 | 0.0548 |  |
| NGTDM Coarseness | -0.2052 | 0.133 |  |
| NGTDM Contrast | -0.2354 | 0.0836 |  |
| NGTDM Busyness | 0.122 | 0.3749 |  |
| NGTDM Complexity | -0.208 | 0.1275 |  |
| NGTDM Strength | -0.1776 | 0.1945 |  |
| NGLCM3D Uniformity mean | -0.2153 | 0.1144 |  |
| NGLCM3D Uniformity max | -0.2371 | 0.0813 |  |
| NGLCM3D Entropy mean | 0.1563 | 0.2546 |  |
| NGLCM3D Entropy max | 0.1423 | 0.3002 |  |
| NGLCM3D Dissimilarity mean | -0.2496 | 0.0661 |  |
| NGLCM3D Dissimilarity max | -0.144 | 0.2943 |  |
| NGLCM3D Contrast mean | -0.2809 | 0.0578 |  |
| NGLCM3D Contrast max | -0.1681 | 0.22 |  |
| NGLCM3D Homogeneity mean | 0.123 | 0.3711 |  |
| NGLCM3D Homogeneity max | 0.0879 | 0.5235 |  |
| NGLCM3D Correlation mean | -0.2449 | 0.0715 |  |
| NGLCM3D Correlation max | -0.2588 | 0.0564 |  |
| NGLCM3DMean Uniformity | -0.1707 | 0.2127 |  |
| NGLCM3DMean Entropy | 0.1206 | 0.3805 |  |
| NGLCM3DMean Dissimilarity | -0.2113 | 0.1216 |  |
| NGLCM3DMean Contrast | -0.1699 | 0.215 |  |
| NGLCM3DMean Homogeneity | 0.1517 | 0.2689 |  |
| NGLCM3DMean Correlation | -0.2599 | 0.0553 |  |
| **GLSZM HighGrayLevelZoneEmphasis** | **0.1795** | **0.1898** | **0.7592** |
| **GLSZM LowGrayLevelZoneEmphasis** | **-0.3599** | **0.007** | **0.028** |
| GLSZM LargeAreaEmphasis | 0.1454 | 0.2894 |  |
| GLSZM SmallAreaEmphasis | -0.1446 | 0.2921 |  |
| GLSZM IntensityVariability | -0.1728 | 0.2072 |  |
| GLSZM RunLengthVariability | -0.1423 | 0.2999 |  |
| GLSZM ZonePercentage | -0.1454 | 0.2894 |  |
| GLRLM ShortRunsEmphasis mean | -0.1331 | 0.3325 |  |
| GLRLM ShortRunsEmphasis max | -0.1066 | 0.4385 |  |
| GLRLM LongRunsEmphasis mean | 0.1532 | 0.2643 |  |
| GLRLM LongRunsEmphasis max | 0.1898 | 0.1652 |  |
| GLRLM GrayLevelNonuniformity mean | -0.151 | 0.2713 |  |
| GLRLM GrayLevelNonuniformity max | -0.1526 | 0.266 |  |
| GLRLM RunLengthNonuniformity mean | -0.1289 | 0.3482 |  |
| GLRLM RunLengthNonuniformity max | -0.1335 | 0.3312 |  |
| GLRLM RunPercentage mean | -0.1557 | 0.2563 |  |
| GLRLM RunPercentage max | -0.1196 | 0.3844 |  |

**Spearman rank correlation test**

Group A

ctHPV16DNA /wbMTV vs. Variable

| Variable | Spearman Rho | p value | Corrected p value |
| --- | --- | --- | --- |
| SUV Histogram Variance | -0.036 | 0.804 |  |
| SUV Histogram Entropy | 0.089 | 0.5388 |  |
| NGLCM Uniformity mean | -0.06 | 0.6787 |  |
| NGLCM Uniformity max | -0.0788 | 0.5867 |  |
| NGLCM Entropy mean | 0.025 | 0.8632 |  |
| NGLCM Entropy max | 0.0145 | 0.9203 |  |
| NGLCM Dissimilarity mean | -0.0858 | 0.5534 |  |
| NGLCM Dissimilarity max | -0.1201 | 0.4063 |  |
| NGLCM Contrast mean | -0.1494 | 0.3005 |  |
| NGLCM Contrast max | -0.1589 | 0.2704 |  |
| NGLCM Homogeneity mean | -0.0037 | 0.9794 |  |
| NGLCM Homogeneity max | -0.0738 | 0.6105 |  |
| NGLCM Inverse Difference Moment mean | -0.0248 | 0.8643 |  |
| NGLCM Inverse Difference Moment max | -0.0481 | 0.7403 |  |
| NGLCM Correlation mean | -0.1024 | 0.4791 |  |
| NGLCM Correlation max | -0.0874 | 0.546 |  |
| NGTDM Coarseness | -0.0643 | 0.6575 |  |
| NGTDM Contrast | -0.093 | 0.5205 |  |
| NGTDM Busyness | 0.1561 | 0.2789 |  |
| NGTDM Complexity | -0.0681 | 0.6384 |  |
| NGTDM Strength | -0.069 | 0.6342 |  |
| NGLCM3D Uniformity mean | -0.0512 | 0.724 |  |
| NGLCM3D Uniformity max | -0.0288 | 0.8427 |  |
| NGLCM3D Entropy mean | 0.0243 | 0.8669 |  |
| NGLCM3D Entropy max | 0.0211 | 0.8844 |  |
| NGLCM3D Dissimilarity mean | -0.0822 | 0.5705 |  |
| NGLCM3D Dissimilarity max | -0.0006 | 0.9968 |  |
| NGLCM3D Contrast mean | -0.1176 | 0.416 |  |
| NGLCM3D Contrast max | -0.0225 | 0.8765 |  |
| NGLCM3D Homogeneity mean | 0.0032 | 0.9826 |  |
| NGLCM3D Homogeneity max | -0.0373 | 0.7968 |  |
| NGLCM3D Correlation mean | -0.0985 | 0.4961 |  |
| NGLCM3D Correlation max | -0.0629 | 0.6645 |  |
| NGLCM3DMean Uniformity | -0.0325 | 0.8226 |  |
| NGLCM3DMean Entropy | 0.0456 | 0.7531 |  |
| NGLCM3DMean Dissimilarity | -0.123 | 0.3948 |  |
| NGLCM3DMean Contrast | -0.1523 | 0.2909 |  |
| NGLCM3DMean Homogeneity | 0.0285 | 0.8445 |  |
| NGLCM3DMean Correlation | -0.0795 | 0.583 |  |
| GLSZM HighGrayLevelZoneEmphasis | 0.0703 | 0.6275 |  |
| **GLSZM LowGrayLevelZoneEmphasis** | **-0.2608** | **0.0674** | **0.2696** |
| GLSZM LargeAreaEmphasis | -0.0137 | 0.9248 |  |
| GLSZM SmallAreaEmphasis | 0.0067 | 0.9633 |  |
| GLSZM IntensityVariability | -0.1065 | 0.4617 |  |
| GLSZM RunLengthVariability | -0.048 | 0.7408 |  |
| GLSZM ZonePercentage | 0.0137 | 0.9248 |  |
| GLRLM ShortRunsEmphasis mean | 0.0549 | 0.705 |  |
| GLRLM ShortRunsEmphasis max | 0.0199 | 0.8909 |  |
| GLRLM LongRunsEmphasis mean | -0.0072 | 0.9604 |  |
| GLRLM LongRunsEmphasis max | 0.0244 | 0.8663 |  |
| GLRLM GrayLevelNonuniformity mean | -0.0911 | 0.5291 |  |
| GLRLM GrayLevelNonuniformity max | -0.0915 | 0.5276 |  |
| GLRLM RunLengthNonuniformity mean | -0.0573 | 0.6927 |  |
| GLRLM RunLengthNonuniformity max | -0.0698 | 0.6301 |  |
| GLRLM RunPercentage mean | 0.0031 | 0.9831 |  |
| GLRLM RunPercentage max | -0.0071 | 0.9609 |  |

Group B

ctHPV16DNA /wbMTV vs. Variable

| Variable | Spearman Rho | p value | Corrected p value |
| --- | --- | --- | --- |
| SUV Histogram Variance | -0.1859 | 0.1741 |  |
| SUV Histogram Entropy | 0.1855 | 0.1752 |  |
| NGLCM Uniformity mean | -0.0846 | 0.539 |  |
| NGLCM Uniformity max | -0.0869 | 0.528 |  |
| NGLCM Entropy mean | 0.008 | 0.9538 |  |
| NGLCM Entropy max | -0.0034 | 0.9803 |  |
| NGLCM Dissimilarity mean | -0.1183 | 0.3895 |  |
| NGLCM Dissimilarity max | -0.0724 | 0.5993 |  |
| NGLCM Contrast mean | -0.1621 | 0.2371 |  |
| NGLCM Contrast max | -0.1177 | 0.392 |  |
| NGLCM Homogeneity mean | 0.0297 | 0.8296 |  |
| NGLCM Homogeneity max | 0.0054 | 0.969 |  |
| NGLCM Inverse Difference Moment mean | 0.001 | 0.9945 |  |
| NGLCM Inverse Difference Moment max | 0.0158 | 0.9089 |  |
| NGLCM Correlation mean | -0.1154 | 0.4014 |  |
| NGLCM Correlation max | -0.1267 | 0.3567 |  |
| NGTDM Coarseness | -0.0612 | 0.6569 |  |
| NGTDM Contrast | -0.0941 | 0.4945 |  |
| NGTDM Busyness | 0.0463 | 0.7369 |  |
| NGTDM Complexity | -0.0709 | 0.607 |  |
| NGTDM Strength | -0.069 | 0.6168 |  |
| NGLCM3D Uniformity mean | -0.0755 | 0.5839 |  |
| NGLCM3D Uniformity max | -0.0962 | 0.4846 |  |
| NGLCM3D Entropy mean | 0.0074 | 0.9573 |  |
| NGLCM3D Entropy max | -0.0003 | 0.9984 |  |
| NGLCM3D Dissimilarity mean | -0.1377 | 0.316 |  |
| NGLCM3D Dissimilarity max | -0.0508 | 0.7125 |  |
| NGLCM3D Contrast mean | -0.1694 | 0.2163 |  |
| NGLCM3D Contrast max | -0.0762 | 0.5805 |  |
| NGLCM3D Homogeneity mean | 0.0129 | 0.9257 |  |
| NGLCM3D Homogeneity max | 0.0157 | 0.9093 |  |
| NGLCM3D Correlation mean | -0.1082 | 0.4317 |  |
| NGLCM3D Correlation max | -0.1159 | 0.3995 |  |
| NGLCM3DMean Uniformity | -0.0546 | 0.6921 |  |
| NGLCM3DMean Entropy | -0.0263 | 0.8486 |  |
| NGLCM3DMean Dissimilarity | -0.0956 | 0.4877 |  |
| NGLCM3DMean Contrast | -0.0601 | 0.6628 |  |
| NGLCM3DMean Homogeneity | 0.0487 | 0.7243 |  |
| NGLCM3DMean Correlation | -0.1181 | 0.3903 |  |
| **GLSZM HighGrayLevelZoneEmphasis** | **0.1804** | **0.1874** | **0.7496** |
| **GLSZM LowGrayLevelZoneEmphasis** | **-0.2887** | **0.0326** | **0.1304** |
| GLSZM LargeAreaEmphasis | 0.0116 | 0.933 |  |
| GLSZM SmallAreaEmphasis | -0.015 | 0.9134 |  |
| GLSZM IntensityVariability | -0.2425 | 0.0745 |  |
| GLSZM RunLengthVariability | -0.2163 | 0.1127 |  |
| GLSZM ZonePercentage | -0.0116 | 0.933 |  |
| GLRLM ShortRunsEmphasis mean | -0.0007 | 0.9957 |  |
| GLRLM ShortRunsEmphasis max | 0.0093 | 0.9464 |  |
| GLRLM LongRunsEmphasis mean | 0.0181 | 0.8956 |  |
| GLRLM LongRunsEmphasis max | 0.0514 | 0.7094 |  |
| GLRLM GrayLevelNonuniformity mean | -0.2219 | 0.1035 |  |
| GLRLM GrayLevelNonuniformity max | -0.224 | 0.1002 |  |
| GLRLM RunLengthNonuniformity mean | -0.2054 | 0.1325 |  |
| GLRLM RunLengthNonuniformity max | -0.2075 | 0.1284 |  |
| GLRLM RunPercentage mean | -0.0183 | 0.8944 |  |
| GLRLM RunPercentage max | -0.0039 | 0.9774 |  |

**Multiple regression analysis**

Group A

Response variable: ctHPV16DNA

R squared = 0.13, p = 0.1683

| Variable | p value | Standardized beta |
| --- | --- | --- |
| SUVmax | 0.3074 | -0.18624 |
| MTV | 0.9447 | 0.014226 |
| wbMTV | 0.7095 | 0.066905 |
| GLSZM LowGrayLevelZoneEmphasis | 0.1396 | -0.25218 |

Group B

Response variable: ctHPV16DNA

R squared = 0.477567, **p <0.0001**

| Variable | p value | Standardized beta |
| --- | --- | --- |
| **HighGrayLevelZoneEmphasis** | **0.0025** | **0.401242** |
| LowGrayLevelZoneEmphasis | 0.207 | 0.178122 |
| SUVmax | 0.3929 | -0.09961 |
| **wbMTV** | **<0.0001** | **0.663854** |
